# The change in groin pain perception after transabdominal preperitoneal inguinal hernia repair with glue fixation: a prospective trial of a single surgeon’s experience

**DOI:** 10.1007/s00464-018-6178-0

**Published:** 2018-03-30

**Authors:** Kryspin Mitura, Karolina Garnysz, Dorota Wyrzykowska, Irmina Michałek

**Affiliations:** 1 General Surgery Department, Siedlce Hospital, ul. Narutowicza 25, 08-110 Siedlce, Poland; 2 Department of Health Sciences, University of Natural Sciences and Humanities, Siedlce, Poland

**Keywords:** Hernia repair, TAPP, Laparoscopic, Quality of life, Preoperative, Pain

## Abstract

**Background:**

Although inguinal hernia is one of the most common conditions requiring surgical treatment, no reliable information is available on the quality of life of patients with inguinal hernias before surgery. Additionally, patients with intense inguinal pain prior to surgery are more susceptible to postoperative chronic pain. In such cases, less invasive laparoscopic techniques can be used, allowing atraumatic mesh fixation to reduce postoperative pain. The aim of the study was to determine, whether these treatments for patients with preoperative pain would minimize upsetting experiences after surgery.

**Materials and methods:**

Data were gathered prospectively from the National Hernia Repair Register for 146 patients who underwent TAPP repairs in the general surgery department (2013–2016). The demographic data were recorded, the pain intensity was determined and patients described the occurrence of pain during ten everyday activities.

**Results:**

The average surgery time was 56.4 min. The follow-up was 23.4 months. The pain before surgery was 4.28 and 12 months after surgery 0.38 (*p* < 0.001). Pain intensity before surgery was scored as 4.1, 4.3, and 4.9 among patients who had a hernia < 12 months, > 1 year, and > 5 years, respectively (*p* = 0.028). Twelve months after surgery, the pain was 0.26, 0.34, and 0.40 (*p* = 0.037), respectively. Patients < 40 years experienced pain before the surgery more often. The intensity of pre-/postoperative pain was significantly higher < 40 years (4.9/0.63) than > 60 years (3.8/0.29).

**Conclusions:**

TAPP inguinal hernia repair with glue fixation significantly decreased the frequency and intensity of the pain compared to that experienced preoperatively. After TAPP repair, the influence of pain on basic everyday activities is substantially lower. Patients under 40 years of age experience frequent and intense pre- and postoperative pain. A longer hernia duration prior to surgery causes increased pre- and postoperative pain.

Although inguinal hernia is one of the most common conditions requiring surgical treatment, little is known regarding changes in the quality of life after hernia repair because no reliable information is available on the quality of life of patients with inguinal hernias before surgery. Most researchers have primarily focused on evaluating postoperative pain, mainly in terms of recurrence and chronic pain [1]. Therefore, whether the pain experienced after surgery influences quality of life compared with preoperative period is unclear. Currently, the simple presence of hernia is considered an indication for surgery [2, 3]. For small asymptomatic hernias, repair may be postponed until symptoms manifest, or the so-called “watchful waiting” [4, 5]. However, the risk associated with hernia incarceration forces surgeons to offer surgical treatment to all patients, regardless of whether the hernia lowers their quality of life. Therefore, a question arises: will quality of life after surgery be lower due to persistent pain in the postoperative period in cases of asymptomatic hernias or hernias with very few symptoms? Additionally, patients with intense inguinal pain prior to surgery are more susceptible to postoperative chronic pain [6, 7]. In such cases, less invasive laparoscopic techniques can be used, allowing atraumatic mesh fixation to reduce postoperative pain [8–10]. These treatments for patients with preoperative pain should minimize upsetting experiences after surgery.

The aim of this work is to compare the pain experienced by patients before and after transabdominal preperitoneal (TAPP) inguinal hernia repair with 3D mesh using glue fixation and its influence on quality of life.

## Materials and methods

Data were gathered prospectively from the National Hernia Repair Register in Poland (KROPP—http://kropp.org.pl), which is an IT tool available to surgeons in Poland that aims at collecting detailed data from patients undergoing surgical treatments. Participation in the register is voluntary. In the database, answers to more than 92 meticulous questions are recorded; participation is mainly dedicated to centers interested in herniology.

The data were collected prospectively from 146 patients who underwent TAPP repairs in the general surgery department within the last 3 years (from 1st Nov 2013 to 31st Oct 2016). The exclusion criteria were of age below 18 years, incarcerated hernia surgery, and conversion to open surgery. The collected data included information regarding patients’ age, sex, occupation, sport activities, hernia duration, incarceration incidents, and hernia size. The patients who reported preoperative pain determined its intensity using the visual analog scale (VAS) and answered additional questions regarding the nature and circumstances of the pain in detail. The patients described the occurrence of pain during ten everyday activities. Fourteen terms describing the nature of the pain were presented to the patients, and each patient chose the term that best described the nature of his pain (sensory description). In addition, the patients selected from six terms describing the emotional character of the pain. The patients marked the frequency of pain and its descriptive intensity at rest and while performing physical activities.

All surgeries were performed under general anesthesia using standard three trocars technique [4]. After reducing the hernial sac and identifying and sufficiently separating the vas deferens and funicular vessels, a folded mesh was placed through the port at the navel. The entire myopectineal orifice was covered with knitted monofilament polypropylene mesh—Surgimesh® XD 11 × 15 cm, density 50 g/m^2^ with average thickness 0.39 mm (Aspide® Medical, La Talaudière, France), which was fixed using Glubran® 2 (GEM Italy, Viareggio, Italy). The peritoneal defect was sutured using V-Loc™ 2-0 (Medtronic, Minneapolis, MN, USA) absorbable sutures. All patients received antibiotic prophylaxis (Cefazolin) and anticoagulants (Enoxaparin sodium). The patients were discharged to home on the first or second day after surgery. One week after the day of the operation, the patients reported to the clinic, where they answered questions regarding pain (VAS). After at least 12 months from the day of the operation, all patients again answered the questions over telephonic interview regarding pain in the groin area and recurrences. The demographic details of the patients are presented in Table 1.

**Table 1:** Demographic details of the patients (*n* = 146)

Demographics	*n*	%
Age	48.9 years (20–72)	
Gender		
Male	132	90.4
Female	14	9.6
BMI	25.4 (20.9–34.6)	
Occupation type		
Sedentary	42	28.8
Moderate physical labor	31	21.2
Heavy physical labor	44	30.1
Retired or unemployed	29	19.9
Sports activity		
Recreational	64	43.8
Professional	13	8.9
None	69	47.3
Hernia duration (months)	14.5 (1–180)	
Hernia reducible		
Yes	136	93.2
No	10	6.8
History of hernia straining		
Yes	11	7.5
No	135	92.5
Pre-op hernia size assessment		
Above the inguinal ligament	65	44.6
Below the inguinal ligament (excluding the scrotal ligament)	57	39.0
Scrotal involvement < 5 cm	18	12.3
Scrotal involvement 5–10 cm	5	3.4
Scrotal involvement > 10 cm	1	0.7
Post-op hernia type		
Direct (M1/M2/M3)	59 (16/31/12)	40.4 (11.0/21.2/8.2)
Indirect (L1/L2/L3)	87 (28/38/21)	59.6 (19.2/26.0/14.4)
Recurrent hernia		
Yes	23	15.7
No	123	84.3

### Data analysis

All data are presented as the means, standard deviations, and percentages. Descriptive statistics were produced for the dataset. The parameter variables were analyzed using ANOVA, and a subgroup analysis was conducted using Student’s *t* test. A *p* value < 0.05 was considered statistically significant.

## Results

Unilateral inguinal hernia repair was performed in 146 patients. The average surgery time was 56.4 min (43–110). Significant intraoperative complications and complications during the period directly after the operation were not observed. In the analyzed material, one case of recurrence was identified, which appeared 11 months after surgery for a large medial hernia (M3). The average follow-up duration was 23.4 months after surgery.

**Fig. 1:**
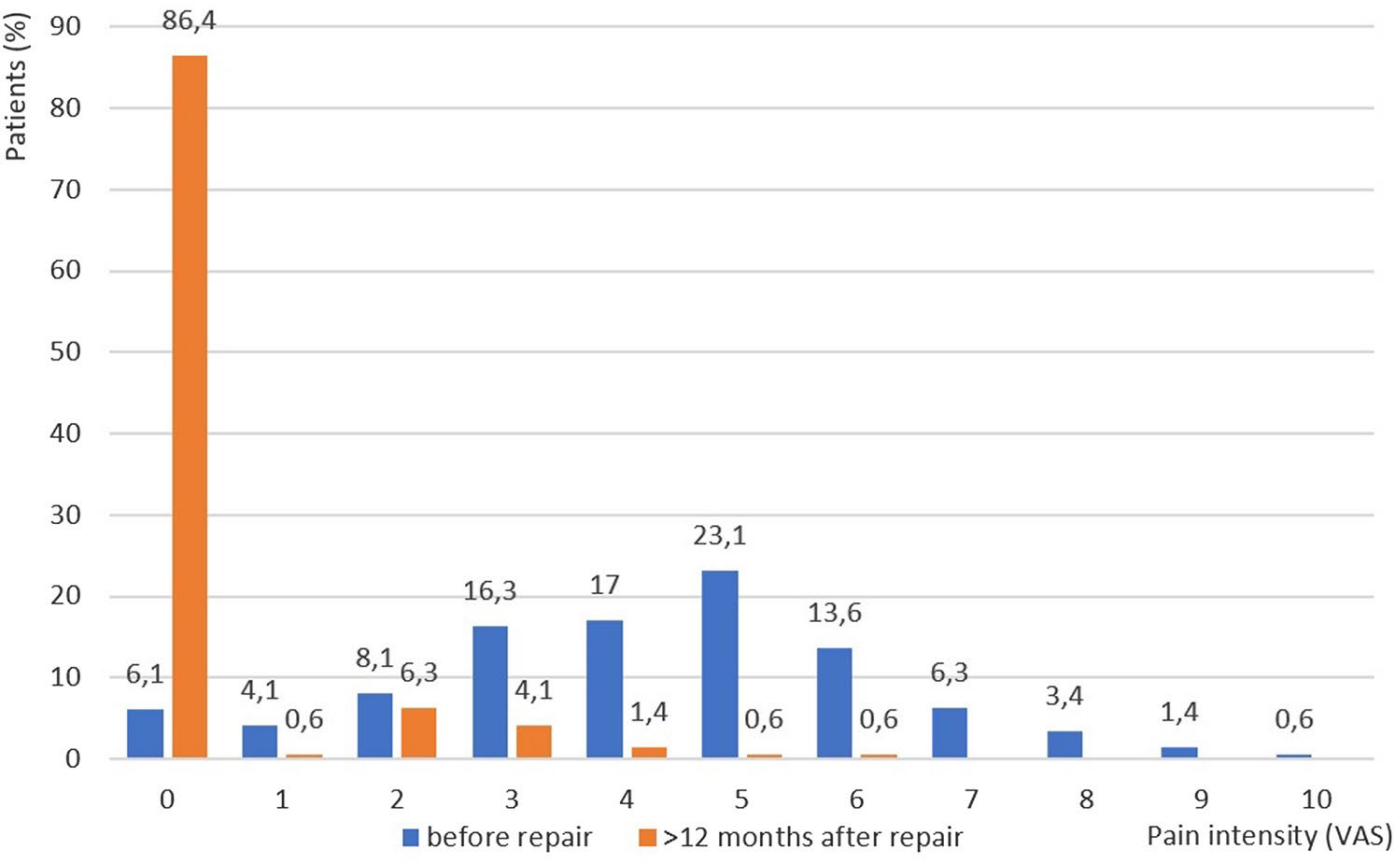
Distribution (%) of pain intensity according to the VAS; pre- and postoperative pain at rest (*n* = 147)

**Fig. 2:**
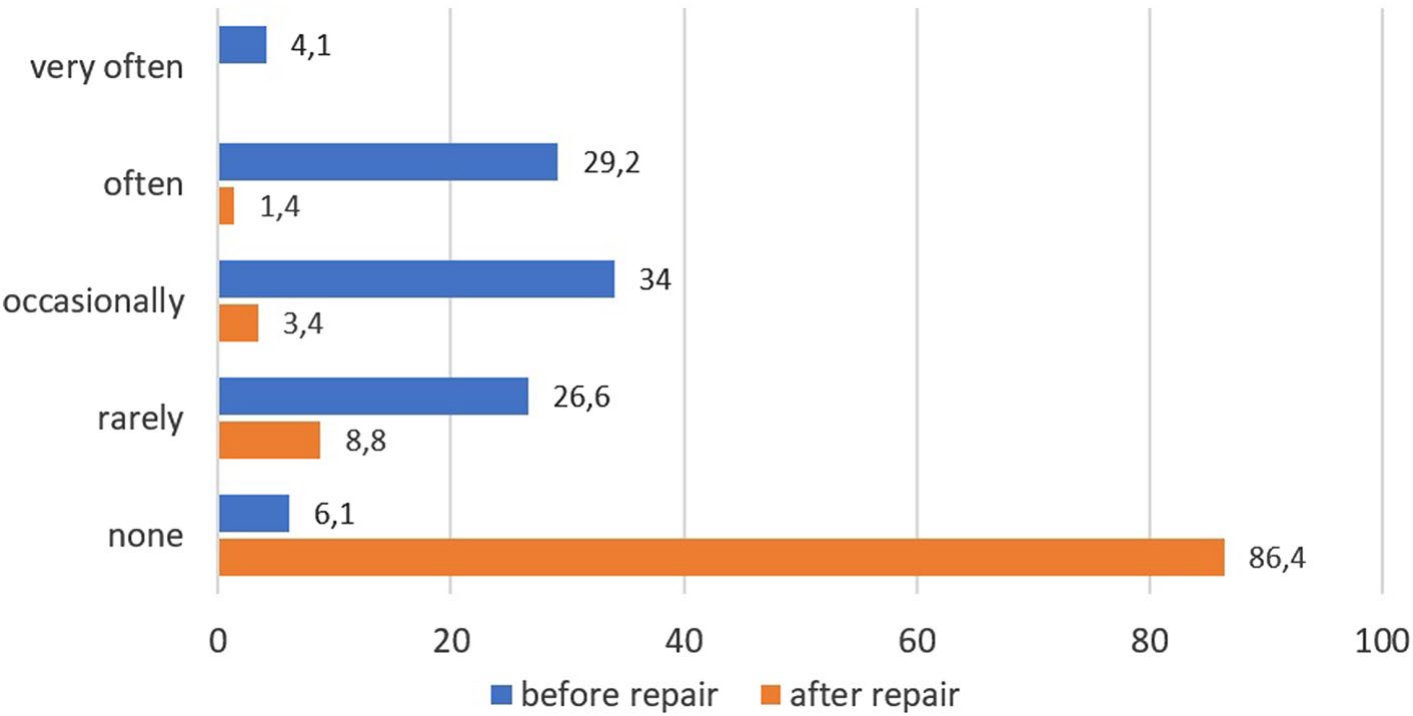
Pain at rest frequency (%) before and after surgery (*n* = 147)

**Fig. 3:**
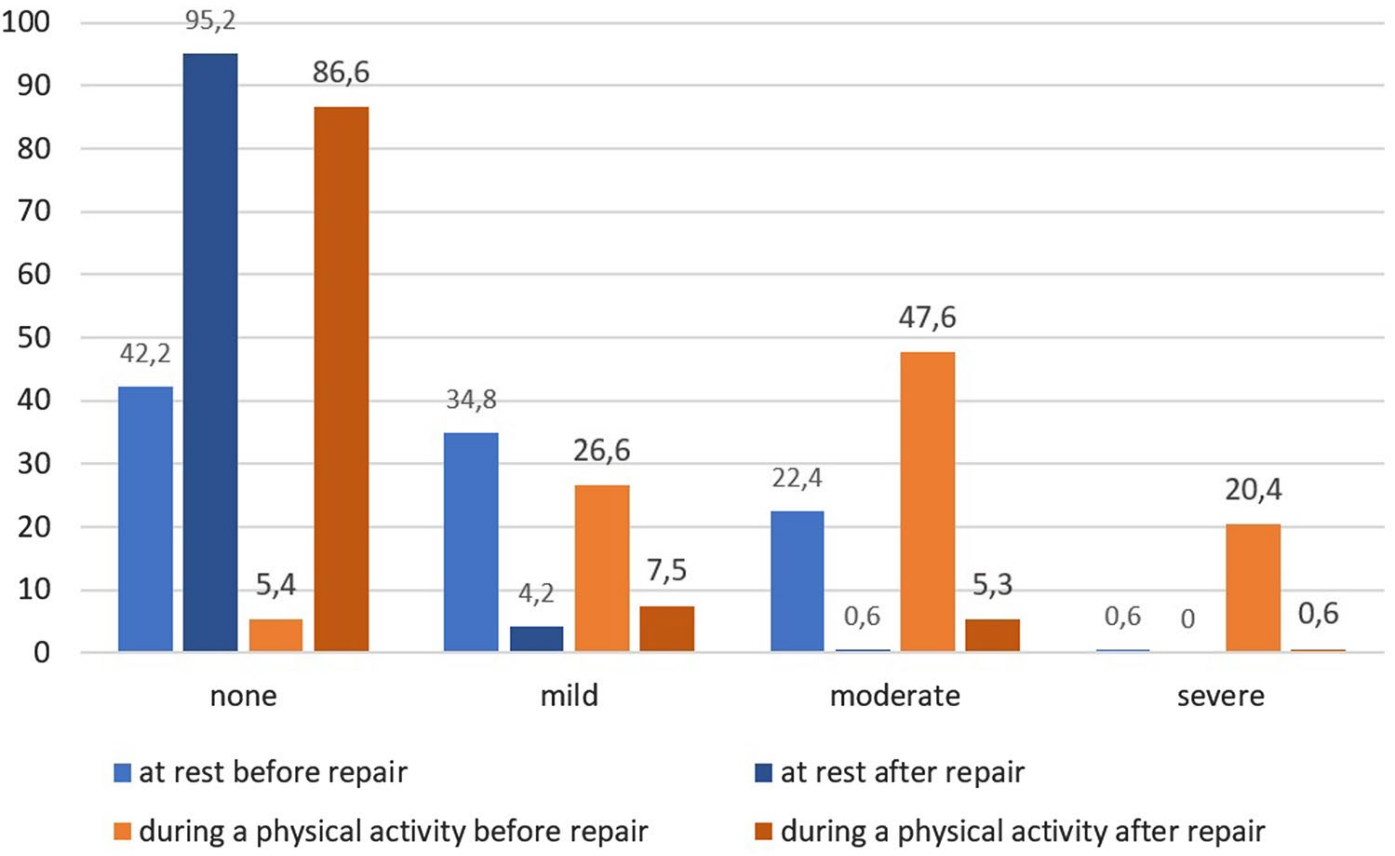
Percentage distribution of patients reporting changes in pain intensity depending on the type of physical activity performed (*n* = 147)

**Fig. 4:**
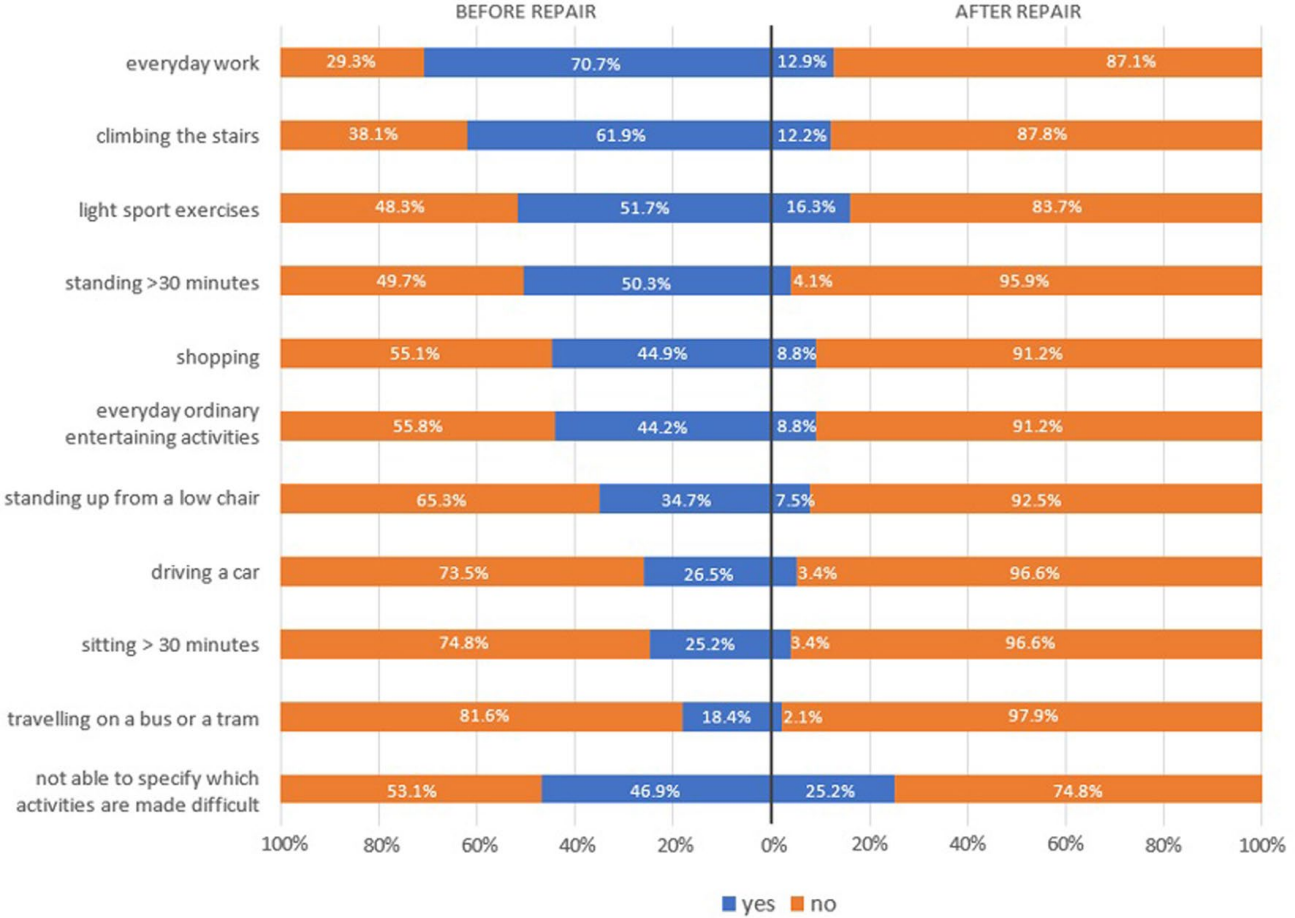
Percentage of patients reporting pain during everyday activities before and after surgery (*n* = 147)

The average intensity of pain at rest according to the VAS prior to surgery was 4.28 (SD 2.16); 7 days after surgery, it was 2.28 (SD 1.79; *p* < 0.001); and 12 months after surgery, it was 0.38 (SD 0.35; *p* < 0.001). Pain intensity at rest according to the VAS and the frequency of pain before and after surgery are presented in Figs. 1 and 2. Changes in pain intensity depending on a performed activity are shown in Fig. 3. Figure 4 presents the influence of pain on daily activities.

BMI differences between the group experiencing preoperative pain at rest (BMI 25.2) and the patients without pain (BMI 25.5) were not observed.

Hernia persistence did not affect the frequency of pain before surgery. In the group of patients who had a hernia for less than 12 months, 61.5% felt pain at rest at some point of the time. A total of 58.5% of the patients who had a hernia for more than a year experienced pain, and 57.3% of the patients who had a hernia for more than 5 years reported pain. Furthermore, gradual increases in pain intensity were noted in these patient groups. In the patients who suffered from pain at rest, its maximum intensity reported at any time before surgery was scored in VAS as 4.1, 4.3, and 4.9 among patients who had a hernia for less than 12 months, for more than a year, and for more than 5 years, respectively, and the differences were statistically significant (*p* = 0.028). Twelve months after surgery, the pain intensity scores were 0.26, 0.34, and 0.40 (*p* = 0.037), respectively.

Patients below 40 years of age experienced pain before the surgery more often (63.3%) than the patients aged 40–60 years (59.2%) and the patients older than 60 years (53.5%). Moreover, the intensity of preoperative pain was significantly higher in the youngest group (VAS 4.9) than that in the middle-aged group (VAS 4.3; *p* = 0.008) and the oldest group (VAS 3.8; *p* = 0.014). Twelve months after the operation, pain intensity scores in these groups were 0.63, 0.41, and 0.29 (*p* = 0.038) for patients younger than 40, aged 40–60, and older than 60 years, respectively.

No serious complications, i.e., life-threatening complications or those requiring another intervention or additional treatment, were observed, including bowel, bladder, or large vessel trauma.

## Discussion

Pain in the groin region can be evaluated on the basis of information collected while taking a patient’s history and during examination. The Short Form 36 (SF-36) questionnaire is a helpful tool for assessing the quality of life; however, its efficiency is limited to the evaluation of chronic disease [11]. Therefore, in 2008, Heniford proposed a new, easy-to-use, and reliable tool for evaluating pain in the inguinal region, i.e., the Carolina Comfort Scale. Using this scale, a patient specifies the intensity of pain during eight daily activities, and the scope of the questions in this scale is consistent with the scope of the questions presented to our study group [12].

Several studies report that patients with inguinal pain undergo surgery; however, the type of pain is often not specified [1, 6, 7]. Additionally, pain is typically addressed, but not its type, frequency, or intensity. Therefore, specifying a group of patients who should generally undergo scheduled surgery on the basis of preoperative pain is impossible. Furthermore, determining a group of patients who could benefit most from surgery and who may suffer from intensified pain and poorer treatment results due to postponing surgery or applying the watchful waiting strategy is also currently impossible because of the lack of a reliable and comprehensive evaluation for preoperative pain in patients with inguinal hernias. Laparoscopic techniques were introduced as effective methods of inguinal hernia repair that could ensure the lowest level of postoperative pain [13]. These techniques minimize the risk of nerve trauma in the groin area and lower the frequency of chronic pain, which has been found to be 3% after open procedures depending on the pain definition used [14, 15]. Unfortunately, laparoscopy fails to prevent the occurrence of pain lasting longer than 3 months and scoring above three points on the VAS in all patients. In our study, 2.6% of the patients defined their inguinal pain as higher than 3 on the VAS. One reason for this category of pain is the development of permanent injuries in inguinal nerve structure due to pressure caused by a hernia [8]. In such cases, pain appears before surgery is performed, and postponing surgery may result in an increased risk of developing chronic pain. Further research may provide new information regarding this matter, and such analyses should therefore be considered. Unfortunately, more and more often, postoperative chronic pain prompts patients to pursue legal action against surgeons [16]. Therefore, determining whether and the extent to which preoperative pain can predict postoperative pain is essential. Our study demonstrated that patients with hernias persisting for more than 12 months experienced more frequent and intense pain.

TAPP laparoscopic repair requires general anesthesia. Usually, an older age at surgery corresponds to a higher risk of requiring perioperative anesthesia according to American Society of Anesthesiologists (ASA). Consequently, some older patients may not qualify for general anesthesia [17]. Therefore, in most studies, a trend is evident: patients undergoing laparoscopic surgery are typically younger than those undergoing open procedures, which was also observed in our study. In addition, we found that younger patients experience preoperative pain more often and with greater intensity. Considering that younger patients are usually more physically and professionally active, performing an operation that allows a quick return to work without long periods of physical disability seems to be the most cost-effective option for this population. Therefore, younger patients may benefit most from laparoscopic surgery.

One method of reducing postoperative pain in patients who undergo laparoscopy is to fix a mesh with glue [18, 19]. Glue is used in areas where a mesh can adhere flat against the flat plane. Therefore, better mesh adherence and less mesh folding can be achieved in the three-dimensional myopectineal orifice with a pre-shaped 3D mesh, which was used in our study. Similar to other researchers, we noticed less intense postoperative pain [18–20]. Moreover, we observed that this method resulted in significantly decreased pain while performing everyday activities compared with that experienced during the preoperative period, which is especially important for younger and physically, socially, and professionally active patients.

## Conclusions

TAPP inguinal hernia repair with glue fixation significantly decreased the frequency and intensity of the pain compared with that experienced preoperatively. After TAPP repair, the influence of pain on basic everyday activities is substantially lower. Patients under 40 years of age experience frequent and intense pre- and postoperative pain. A longer hernia duration prior to surgery causes increased pre- and postoperative pain.
